# Study on Blending of Wall Material of the Nonel Tube by CSW/PE-g-MAH

**DOI:** 10.1155/2019/7590692

**Published:** 2019-12-23

**Authors:** Hong-wei Li, Bin-bin Zhang, Ji-nian Yang, Huan Li, Ji-chang Gui, Zhan Lei

**Affiliations:** ^1^School of Chemistry and Chemical Engineering, Anhui University of Science and Technology, Huainan 232001, Anhui, China; ^2^School of Material Science and Engineering, Anhui University of Science and Technology, Huainan 232001, Anhui, China

## Abstract

In order to improve the strength and resistance of ordinary nonel tubes, calcium sulfate whiskers (CSW, treated with silane coupling agent) and maleic anhydride grafted polyethylene (PE-g-MAH) are used to control the wall material of the nonel tube that the blending of the low-density polyethylene was enhanced. The effects of mass fraction of CSW or PE-g-MAH on the tensile properties, interfacial structure, melting and crystallization characteristics, and thermal decomposition behavior of the composite system were studied, and the thermal decomposition kinetics were calculated. The results show that, relative to pure LDPE, the strength of LDPE/CSW (85/15) is increased by 7.58%, and the strength of LDPE/CSW/PE-g-MAH (84/15/1) is increased by 7.58%. The addition of CSW or PE-g-MAH has gradually changed the fracture mode of the LDPE matrix. Thermal analysis shows that CSW can reduce the crystallinity of LDPE. The melting and crystallization characteristics of LDPE/CSW/PE-g-MAH composites have little effect, but the thermal decomposition stability is improved. The kinetic analysis showed that the reaction order (*n*) was around 1, CSW could improve LDPE/CSW thermal decomposition activation energy, and PE-g-MAH increased the thermal decomposition activation energy of LDPE/CSW/PE-g-MAH.

## 1. Introduction

Ordinary nonel tube has excellent ignition characteristics. The whole blasting network can be triggered by electric spark, which avoids the requirement of electric network for initiating current [1, 2]. The detonation method of the nonel tube was observed under high-speed cameras [3, 4], and the design and connection method of the blasting network was widely studied and applied [5–8]. The source material of ordinary plastic nonel tube is low-density polyethylene (LDPE), which has the characteristics of good toughness, easy processing, and wide application range [9–13]. With the development of the civil explosive industry, the use of the existing ordinary nonel tube is limited, and new requirements are imposed on the strength and heat resistance of the nonel tube.

For decades, people's research on detonating tubes has focused on the study of high-intensity and high-precision and supporting detonators. The core of the research was the delay accuracy of nonel tubes [14–16]. There was also research on the color-changing nonel tube to explore the discoloration performance of elements such as aluminum and chlorine in the nonel tube [17, 18]. The purpose of these studies was to improve the strength and heat resistance of the nonel tube and to increase the range of use of the nonel tube. In view of this, this paper intends to use the basic research on the material of the nonel tube to explore the blending modification of LDPE, to prepare a low-cost high-strength low-density polyethylene nonel tube, and to improve the scope of use of plastic nonel tube.

Calcium sulfate whiskers (CSW) were artificial crystals, which were commonly used to modify the strength of composites [19–23]. For example, in the study of the mechanical properties of CW-enhanced modification, the modified CSW has a strong interfacial bond with the PVC matrix [22], and CSW could effectively improve the thermal stability of the material [23]. Maleic anhydride grafted polyethylene (PE-g-MAH) is a compatibilizer commonly used to increase the compatibility of the infused system and improve the physical and chemical properties of the matrix [24–28]. For example, PE-g-MAH not only improved the thermal stability of LDPE/ESP composites, but also improved its interfacial bonding [26]. The addition of PE-g-MAH also improved tensile strength and Young's modulus of the LDPE/NR/WHF composites [27]. However, there are few reports on the blending of LDPE/CSW/PE-g-MAH. In this paper, based on the strength of CSW-enhanced LDPE, the strength is increased again by adding the compatibilizer PE-g-MAH. By observing the cross-sectional morphology and melt crystallization properties of LDPE/CSW/PE-g-MAH composites and analyzing their thermogravimetric losses, the apparent activation energy was calculated and compared by Kissinger [29] and Carrasco et al. [30, 31], who provided a reference for obtaining a material with high strength and good heat resistance.

## 2. Experimental

### 2.1. Raw Materials

LDPE (1I2A-1, China Petrochemical Co., Ltd. Beijing Yanshan Branch, China) is a pellet with a density of 0.92 g/cm^3^ and a melt flow rate (MFR) of 2.0 g/10 min. Powders of CSW (white, Tang Brothers Technology Co., Ltd, Wuhan, China) have a density of 2.69 g/cm^3^ and a length-to-diameter ratio of 30 to 70, which is a crystal form of calcium sulfate hemihydrate CaSO4·0.5H_2_O. Silane coupling agent, KH550, Nanjing Dawn Chemical Co., Ltd., analysis pure. The compatibilizer PE-g-MAH (manufactured by Dow, USA) is a pellet with a melt flow rate (MFR) of 2.5 g/10 min and a density of 0.95 g/cm^3^. Reagents such as anhydrous ethanol, silicone oil, and liquid paraffin are of analytical grade and are commercially available.

### 2.2. Preparation of Samples

According to the literature [32], in order to improve the interface compatibility between CSW and LDPE, the surface modification of CSW with KH550 was carried out.

An appropriate amount of LDPE, CSW, and PE-g-MAH was weighed and dried under vacuum at 60°C for 24 hours. A high-speed mixer (SHR-10A, Zhangjiagang Jianguo Machinery Co., Ltd) is used for premixing according to the ratio shown in Table 1 and is subjected to melt extrusion through a double-screw extruder (SHJ-20, Nanjing Jaya Co., Ltd), and water-cooled granulation, and the temperature of I ∼ VI temperature zones was 185°C, 190°C, 195°C, 190°C, 195°C, and 185°C, and the cooling water temperature was 17°C. The new material was again vacuum-dried at 60°C for 24 h and then injection molded (170–200°C) into a strip in a vertical injection molding machine (FT-200, Fomtec Machinery Co., LTD, Suzhou, China) (GB/T 1040.2-2006, 1A), standard dumbbell type.

### 2.3. Measurements

#### 2.3.1. Mechanical Tests

Each sample was measured prior to the uniaxial tensile test. The span length test of the standard dumbbell type test was 62 mm, the length was 80 mm, the width was 10 mm, and the thickness was 4 mm. The uniaxial tensile test was carried out according to the Chinese Standard GB/T 1040.1-2006 on a universal electronic testing machine (WDW-50, Shenzhen Kaiqiangli Co., LTD, China) at a rate of 50 mm/min. The gauge length (type 1A) of the dumbbell-shaped specimen was 50 mm, and the impact test was performed on a TCL-25J impact tester (Tanhor Co., LTD, Jilin, China). The impact test results directly give the impact energy of each sample. The elastic modulus, elongation at break, tensile strength, and other values of the sample are obtained by an auxiliary computer, and the strain-stress curve is integrated to obtain the tensile toughness. All test results are taken from the average of 5 sets of samples and are carried out at room temperature.

#### 2.3.2. Scanning Electron Microscopy (SEM)

A sample with higher quality was selected, and the sample was transferred to a bucket containing liquid nitrogen using a wooden stick and frozen for 20 minutes. The sample was taken out and the brittle fracture treatment was carried out using the brittle cutting machine, the sample was collected, the section should not be touched by hand, and 4 *∗* 4 mm small pieces were taken at the section. The brittle fracture section was subjected to Au spray treatment, and then the cross-sectional morphology was observed by field emission scanning electron microscopy (FESEM, JSM 7600F, JEOL Ltd., Japan).

#### 2.3.3. Differential Scanning Calorimeter (DSC)

 Nonisothermal melting and cooling curves of the samples were obtained using a differential scanning calorimeter (DSC, Q200, TA, USA). The temperature rise and fall rate of the DSC was 10°C/min, and the reaction was carried out under an N_2_ atmosphere at a concentration of 60 mL/min. The temperature was raised from room temperature to 180°C for 10 min and then lowered to room temperature.

#### 2.3.4. Thermogravimetric Analysis (TGA)

The mass loss and mass loss rate curves were obtained using a thermogravimetric analyzer (TGA, Q500, TA, USA) and the temperature was raised from room temperature to 700°C. The amount of sample in the alumina crucible was about 15 mg, in an N_2_ atmosphere at a flow rate of 60 mL/min, and the temperature was raised at a rate of 10°C/min.

## 3. Results and Analysis

### 3.1. Mechanical Properties

**Figure 1:**
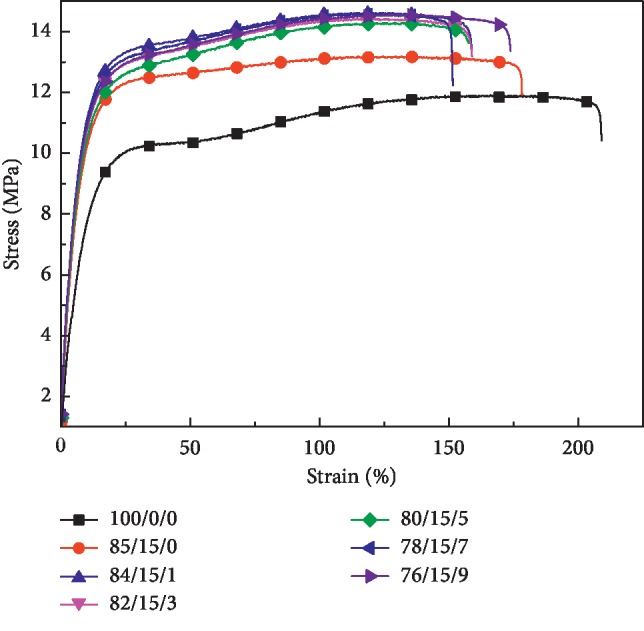
Stress-strain curve of LDPE/CSW/PE-g-MAH composites.

The stress-strain curves of LDPE/CSW/PE-g-MAH composites are shown in Figure 1, and other mechanical properties are shown in Table 2. The tensile strength, elastic modulus, and elongation at break of the material correspond to their strength, stiffness, and toughness [33–35]. Figure 1 shows that, after adding CSW, the strength and stiffness are improved. After adding PE-g-MAH, the strength and stiffness are increased again, but as the mass fraction of PE-g-MAH increases, the increase in the tensile strength and modulus of elasticity of the composite material was less. The introduction of CSW showed that the matrix fracture failure mode of LDPE changed, and the reintroduction of PE-g-MAH increased the compatible phase of LDPE/CSW interface and strengthened the fracture failure mode of 85/15/0. The tensile toughness calculated according to the method of [36] is shown in Table 2 and is substantially consistent with its toughness and is reduced. The addition of PE-g-MAH increased the tensile strength and Young's modulus of LDPE/NR/WHF composites, and the tendency of elongation at break was consistent [27].

The stiffness of LDPE/CSW/PE-g-MAH composites increased by 75.93%∼93.79%, that is, the resistance to elastic deformation was enhanced effectively, ensuring that the nonel tube is not subjected to artificial pressure to cause the powder to fall off and gather. After adding 15% CSW to LDPE, the 85/15/0 strength increased by 7.58% (0.91 MPa), with the addition of PE-g-MAH with mass fraction of 1%, 3%, 5%, 7%, and 9%, the strength increased by 18.90%∼23.14% (2.27∼2.78 MPa), and the strength increased significantly. It is not difficult to find that by adding only 1% PE-g-MAH, the 84/15/1 strength can be increased by 19.98% (2.4 MPa), and for the preparation of the nonel tube, the heat resistance and production cost of the material should also be considered, but the addition amount of PE-g-MAH should not exceed 5%.

### 3.2. Microstructure

**Figure 2:**
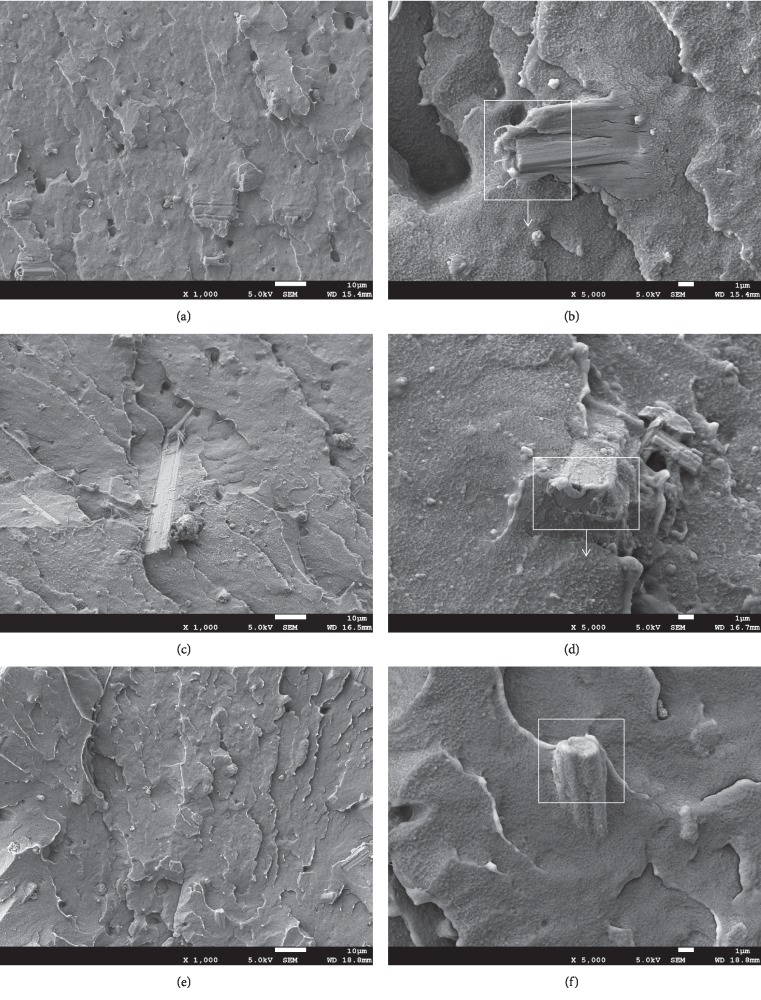
Microstructures of LDPE/CSW/PE-g-MAH composites: (a)∼(b) 85/15/0, (c)∼(d) 84/15/1, and (e)∼(f) 80/15/5.

Figure 2 shows an SEM image of the impact profile of LDPE/CSW/PE-g-MAH composites after cryogenic brittle fracture of liquid nitrogen. It can be seen from the visible whiskers and the pores left after the detachment that the CSW or PE-g-MAH is evenly distributed in the cross section of the sample, and no obvious agglomeration occurs. Under high magnification, the 85/15/0 composite material and the 84/15/1 and 80/15/5 composite materials have obvious differences in cross section. The interface between the CSW and the LDPE matrix was very tight, and the exposed whisker surface was covered with a layer of resin adhering matter, and the pores scattered around it showed obvious irregularities.  Figure 2(b) shows that the fracture process of the material is not completely in the matrix, and there is a large area of exposed whisker interface, and it is proved that the process of breaking the CSW and LDPE has the dissociation of the interface between CSW and LDPE in addition to the tear of the matrix; it is consistent with the increase in 85/15/0 strength in the mechanical properties, as shown in Table 2. The addition of 1% PE-g-MAH, such as the existence of (d) diagram compatibilizer PE-g-MAH, will reduce the exposed whisker interface in the cross section. Combined with the strength and stiffness properties of 84/15/1 in Figure 1 and Table 2, the dissociation is weak at this time, and the matrix tear is the main failure mode of the composite; it is consistent with the conclusion that PE-g-MAH can change the interface bonding property of composites in [26]. As shown in Figure 2(f), the whiskers of the 80/15/5 section are tightly covered by the LDPE resin adherend, and the fracture process has substantially no interface dissociation, which is completely the matrix tear. The main reason for this is that in addition to the organic functionalization of the surface of the CSW by the interface coupling agent, it has good interfacial compatibility with the LDPE matrix, and it also benefits from the compatibilization of PE-g-MAH, which makes the compatibility of the interface between CSW and LDPE complete, which is very beneficial to improve the mechanical properties of the composite.

### 3.3. Melting and Crystallization

**Figure 3:**
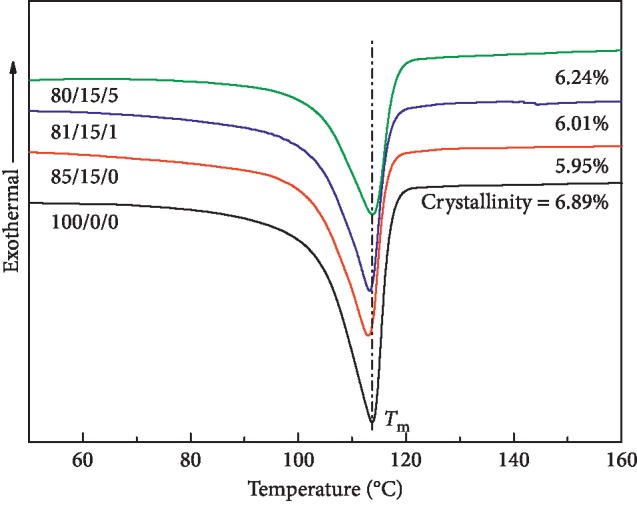
DSC heating curve of LDPE/CSW/PE-g-MAH composites.

Through the characterization of mechanical properties and cross section morphology, the LDPE/CSW/PE-g-MAH ratio component with improved strength is obtained. However, as an important material for detonating tube wall, heat resistance is also an important standard in addition to strength, which is also an important standard.  Figure 3 shows the DSC heating curve of LDPE/CSW/PE-g-MAH composites. According to the melt-crystallization enthalpy (Δ*H*_m_) of 23.75 J/g, when LDPE is completely crystallized [36], the crystallinity (*X*_c_) of the composite material and the melting temperature peak temperature (*T*_m_) of LDPE can be calculated and are shown in Table 3. The *T*_m_ of 85/15/0 has decreased, and on the basis of it, 84/15/1 and 80/15/5 have been improved, but the *T*_m_ range of all samples is 113.06∼113.88°C, and the fluctuation range is small. It indicates that the introduction of CSW or PE-g-MAH has little effect on the *T*_m_ of LDPE/CSW/PE-g-MAH composites.

**Figure 4:**
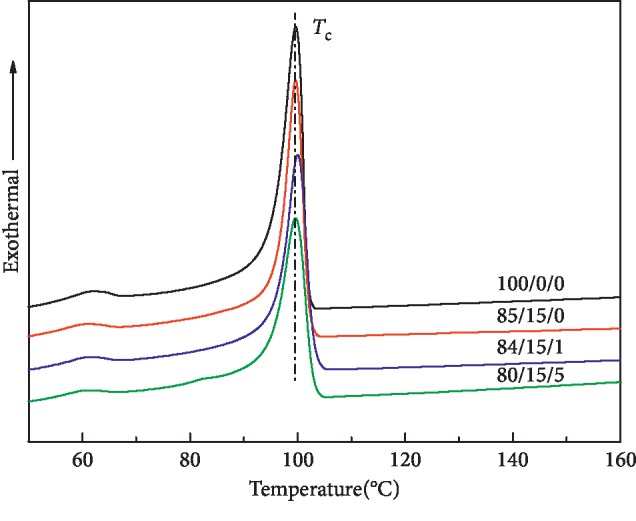
DSC cooling curve of LDPE/CSW/PE-g-MAH composites.

Figure 4 shows the DSC cooling curve of the LDPE/CSW/PE-g-MAH composite material, and the crystallization peak temperature (*T*_c_) is shown in Table 3. The temperature of *T*_c_ is between 99.06 and 100.2°C, and the fluctuation range is also small, which has little effect on it. The trend of crystallinity was consistent with *T*_m_. The combination may be that PE-g-MAH hindered the blocking effect of CSW on crystallization of LDPE, and the overall crystallinity of LDPE/CSW/PE-g-MAH increased. In general, from the melting and crystallization properties of LDPE/CSW/PE-g-MAH composites, either CSW or PE-g-MAH has little effect on LDPE. However, there are no restrictions on the melting and crystallization properties of LDPE.

### 3.4. Thermal Decomposition Characteristics

**Figure 5:**
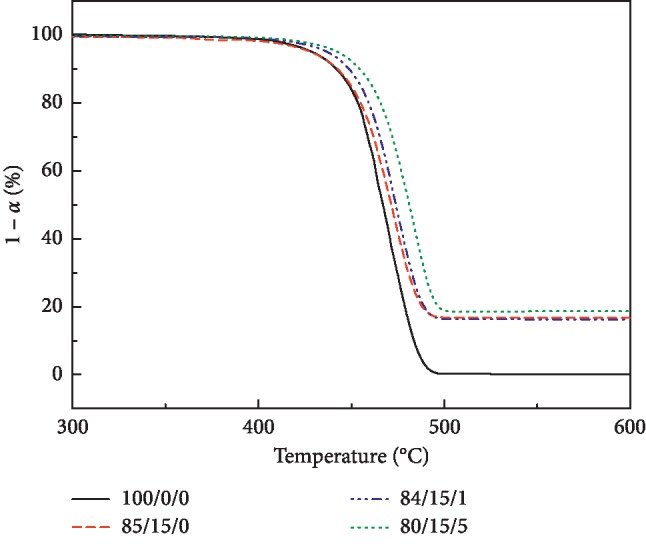
TG curve of LDPE/CSW/PE-g-MAH composites.

Figure 5 shows the TG curve of LDPE/CSW/PE-g-MAH composite, and the initial weight loss temperature (*T*_o_) (5% weight loss of TG), 600°C weight loss residual mass fraction (*W*_600_), and final weight loss residual (Char (%))are shown in Table 4. CSW or PE-g-MAH had a constant weight in weight loss at 600°C and higher because CaSO_4_·0.5H_2_O in CSW decomposes CaSO_4_ at high temperature. In addition, *T*_o_ of the 85/15/0 was lowered by 0.67°C with respect to 100/0/0 because the H_2_O molecules of CaSO_4_·0.5H_2_O in CSW were decomposed in the low-temperature region. *T*_o_ of 85/15/0 and 80/15/5, respectively, moved 8.16°C and 13.81°C to the high-temperature zone. This is because the weight loss temperature of CSW or PE-g-MAH is higher than that of LDPE, which improves LDPE/CSW/PE-g-MAH composite heat resistance.

**Figure 6:**
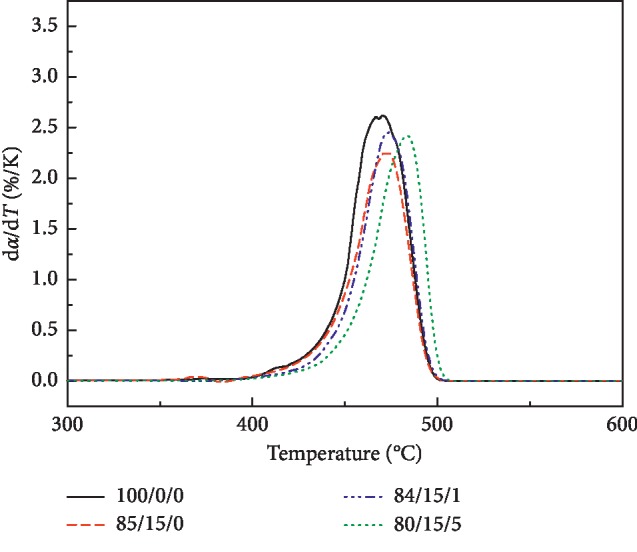
DTG curve of LDPE/CSW/PE-g-MAH composites.

Figure 6 shows the DTG curve of the LDPE/CSW/PE-g-MAH composite. The maximum decomposition temperature *T*_p_, the conversion rate *α*_p_ (%), and the maximum decomposition rate (d*α*/d*T*)_p_ (%/K) are shown in Table 4. The addition of CSW or PE-g-MAH shifted the *T*_p_ of DTG of LDPE/CSW/PE-g-MAH composites to 2.64∼13.09°C in the high-temperature region and decreased (d*α*/d*T*)_p_ by 0.163–0.363%/K. *α*_p_ decreased by 3.21∼10.93%, and the fluctuation was significant, indicating that the thermal decomposition stability of LDPE/CSW/PE-g-MAH composites was improved. Considering the economic cost of enhanced modification, combined with the mechanical properties of 84/15/1, the amount of PE-g-MAH should be higher than 1%, but not 5%, because the cost of 80/15/5 will increase a lot. Because the cost of 80/15/5 will increase a lot, considering its mechanical and thermal properties, it is not as good as 84/15/1.

### 3.5. Thermal Decomposition Kinetics Calculation

**Figure 7:**
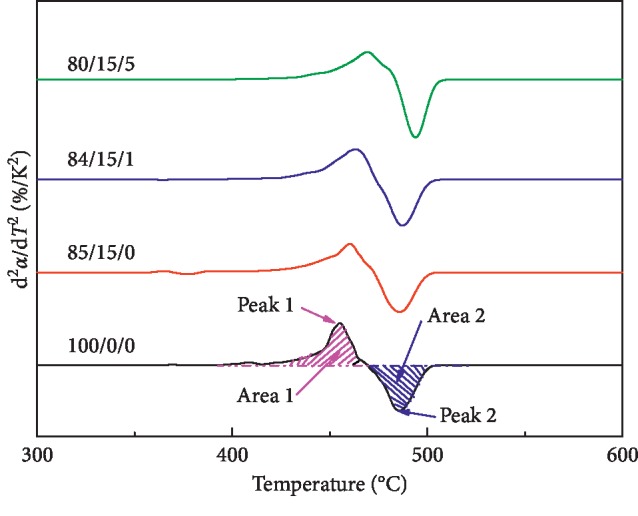
Variation of 2nd conversion derivative as a function of temperature for investigated samples.

Figure 7 shows the first derivative curve of the mass loss rate of LDPE/CSW/PE-g-MAH composites, reflecting the trend of d^2^*α*/d*T*^2^ with temperature (*T*). Theoretically, Kissinger [29] and Carrasco et al. [30, 31] first calculate the reaction order (*n*), and the apparent activation energy *E* (kJ/mol) of LDPE/CSW/PE-g-MAH composites was calculated according to the following equation:

(1) $$\begin{array}{c} E=nRT_{\text{p}}^{2}\frac{{\left(\text{d}\alpha /\text{d}T\right)}_{\text{p}}}{\left(1-\alpha_{\text{p}}\right)}, \end{array}$$

where *R* is the gas constant that is equal to 8.314 J/(mol K), (d*α*/d*T*)_p_ is the reaction rate at the temperature of *T*_p_ and from DTG curves, and *α*_p_ is the reaction conversion rate at the temperature of *T*_p_ and from TG curves.

The core of the calculation of the above two methods lies in the method of *n*. The Kissinger method mainly obtains *S* by the peak ratio, that is, *S* is equal to peak 1 and peak 2, *S* is a shape factor, and then *n* is calculated according to the following equation:

(2) $$\begin{array}{c} n=1.26\cdot \sqrt{S}. \end{array}$$

However, the Carrasco method directly obtains *n* according to the ratio of the area, which is expressed in equation (2):

(3) $$\begin{array}{c} n=\frac{\int_{i_{\left(\alpha =0\right)}}^{T_{\text{p}}}{\left({\text{d}}^{2}\alpha /\text{d}T^{2}\right)}_{\text{area }1}\cdot \text{d}T}{\int_{p}^{T_{\text{f}\left(\alpha =\max\right)}}{\left({\text{d}}^{2}\alpha /\text{d}T^{2}\right)}_{\text{area }2}\cdot \text{d}T}, \end{array}$$

where (d^2^*α*/d*T*^2^) is the rate of change of the reaction rate.

According to the detailed analysis of the literature, the calculation method of equation (4) is generally not used to obtain *n* [30, 31, 37] because *n* calculated by this method is a variation, so Kissinger and Carrasco are used, and the calculation of related parameters is performed. The results are shown in Table 5:

(4) $$\begin{array}{c} n=\frac{\ln\left[{\left(\text{d}\alpha /\text{d}T\right)}_{\text{p}}/\left(\text{d}\alpha /\text{d}T\right)\right]}{\left\{\left[T_{\text{p}}\cdot \left(T_{\text{p}}-T\right)\cdot {\left(\text{d}\alpha /\text{d}T\right)}_{\text{p}}\right]/\left[T\cdot \left(1-\alpha_{\text{p}}\right)\right]\right\}-\ln\left[\left(1-\alpha \right)/\left(1-\alpha_{\text{p}}\right)\right]}. \end{array}$$

The range of *n* obtained by Kissinger is 0.954 to 1.092, and the range of *n* obtained by Carrasco is 0.917 to 1.041, and the overall difference is about 0.5, but the whole is also fluctuating around 1.00. The source of this error was related to the calculation method. *S* is only related to the peak value of the curve, but not related to the long or wide shape of the curve peak. That is to say, the use of *S* for *n* is lacking. The other reason is the error. It has been enlarged by 1.26 times and is usually better than the Carrasco method [37].

From Table 5, it is easy to find that the trend of *n* change obtained by the two methods of Kissinger and Carrasco is inconsistent, but the trend of the last calculated *E* is consistent. The addition of CSW will reduce the *E* of LDPE, which is consistent with the general rule of material blending. The inorganic material CSW usually reduces the *E* of the composite [37]. The addition of PE-g-MAH caused the *E* of LDPE/CSW to rise, but the increase of *E* was small. It may be that PE-g-MAH is caused by low molecular weight and small internal molecular chain. In combination with the Kissinger method, the *E* of 85/15/0 is 234.37 kJ/mol, the *E* of 84/15/1 is 239.37 kJ/mol, and the *E* of 80/15/5 is 246.95 kJ/mol. The span of *E* is small, but compared with the amount of PE-g-MAH added, it is also obvious. In addition, the span of *E* calculated by the Carrasco method is larger than that by the Kissinger method, but the *E* of 100/0/0 is 266.68 kJ/mol, which is less than the 317.66 kJ/mol (Kissinger method), and the coefficient of variation (CV) of the two methods is 0.0569 and 0.0524, respectively. The reason for the small difference is that there is a blending study of the three materials, and the properties of the composite material fluctuate greatly, but it still shows that the Carrasco method is more reliable.

In summary, the *E* reduction of LDPE/CSW/PE-g-MAH composites does not indicate a decrease in thermal decomposition stability. Because the *E* was calculated as *T*_p_, the reaction progress *α*_p_ was less than 50%, and the thermal decomposition stability of the composite could not be directly judged. However, the addition of PE-g-MAH can increase *E*, which is beneficial to the *E* increase of LDPE/CSW/PE-g-MAH composites, and increases the thermal decomposition stability of the composite.

## 4. Conclusion

Through mechanical properties testing, adding 15% CSW to LDPE can increase its strength by 7.58%, the addition of a small amount of compatibilizer increased the strength of the LDPE/CSW/PE-g-MAH composite, and the strength of 85/15/1 is 19.98% higher than that of pure LDPE. It has a positive significance for the strength increase of the nonel tube.

Through the observation under a high power microscope, it was found that the way of fracture changed. After adding 15% CSW to LDPE, the fracture mode was the coexistence of matrix tear and interface dissociation. With the addition of PE-g-MAH, the interface dissociation was limited. When the content of PE-g-MAH reaches 5%, the fracture mode of the interface is only the matrix fracture. It is well explained that the mechanical properties of LDPE/CSW/PE-g-MAH composites will be significantly improved in two stages.

The addition of CSW or PE-g-MAH has little effect on the melting and crystallization properties of the composite, but it has a certain improvement on the thermal decomposition stability and the *T*_o_ of 85/15/1 and 80/15/5 moved 8.16°C and 13.81°C, respectively, to the high-temperature zone. The addition of CSW or PE-g-MAH causes the *T*_p_ of the DTG of the LDPE/CSW/PE-g-MAH composite to move to the high temperature range of 2.64∼13.09°C. (d*α*/d*T*)_p_ decreased by 0.163–0.363%/K, *α*_p_ decreased by 3.21∼10.93%, and the fluctuation was significant, indicating that the thermal decomposition stability of LDPE/CSW/PE-g-MAH composites was improved. Through the two methods of Kissinger and Carrasco kinetic calculations, it is proved that PE-g-MAH can increase the *E* of the composite material, and the above conclusions are supported.

## Figures and Tables

**Table 1:** Mass fraction ratio of LDPE/CSW/PE-g-MAH composites.

Sample	LDPE (%)	CSW (%)	PE-g-MAH (%)
100/0/0	100	—	—
85/15/0	85	15	—
84/15/1	84	15	1
82/15/3	82	15	3
80/15/5	80	15	5
78/15/7	78	15	7
76/15/9	76	15	9

**Table 2:** Mechanical properties of LDPE/CSW/PE-g-MAH composites.

Sample	Tensile strength (MPa)	Elastic modulus (MPa)	Elongation at break (%)	Tensile toughness (MJ/m^3^)
100/0/0	12.01	103.57	203.04	22.64
85/15/0	12.92	158.98	167.53	22.24
84/15/1	14.41	182.21	150.92	21.64
82/15/3	14.43	191.20	152.30	21.25
80/15/5	14.28	180.55	158.83	21.09
78/15/7	14.61	200.14	149.84	20.46
76/15/9	14.79	199.62	175.20	21.54

**Table 3:** Melting and crystallization parameters of DSC curves of LDPE/CSW/PE-g-MAH composites.

Samples	*T*_c_ (°C)	*T*_m_ (°C)	Δ*H*_m_ (J/g)	*X*_c_ (%)
100/0/0	99.66	113.68	15.65	3.89
85/15/0	99.06	113.06	13.52	5.95
84/15/1	100.02	113.27	13.64	6.01
80/15/5	99.56	113.88	14.16	6.24

Δ*H*_m_ is corrected for the percentage of LDPE phase in the composites.

**Table 4:** Thermal degradation parameters for LDPE/CSW/PE-g-MAH composites.

Sample	*T*_o_ (°C)	*T*_p_ (°C)	*α*_p_ (%)	(dα/dT)_p_ (% (°C))	*W*_600_ (%)	Char (%)
100/0/0	428.92	470.34	58.66	2.616	0.17	0.03
85/15/0	428.25	473.33	54.35	2.253	16.69	16.57
84/15/1	437.08	472.98	47.73	2.453	16.27	16.21
80/15/5	442.73	483.43	55.45	2.421	18.67	18.51

**Table 5:** Reaction order (*n*) and activation energy (*E*) for LDPE/CSW/PE-g-MAH composites calculated by Kissinger and Carrasco methods.

Samples	Kissinger			Carrasco	
	*s*	*n*	*E* (KJ/mol)	*n*	*E* (KJ/mol)
100/0/0	0.752	1.092	317.66	0.917	266.68
85/15/0	0.662	1.025	234.37	0.941	215.16
84/15/1	0.765	1.102	239.37	1.041	226.12
80/15/5	0.573	0.954	246.95	0.921	238.41
		C. V = 0.0569		C. V = 0.0524	

## Data Availability

The data that are used to support the findings of this study are available from the first author upon reasonable request.
